# Use of auxiliary devices during retreatment of direct resin composite veneers

**DOI:** 10.1371/journal.pone.0252171

**Published:** 2021-06-16

**Authors:** Fabrício Daniel Finotti Guarnieri, André Luiz Fraga Briso, Fernanda de Souza e Silva Ramos, Lara Maria Bueno Esteves, Érika Mayumi Omoto, Renato Herman Sundfeld, Ticiane Cestari Fagundes

**Affiliations:** Department of Preventive and Restorative Dentistry, School of Dentistry, São Paulo State University (UNESP), Araçatuba, SP, Brazil; University Lyon 1 Faculty of Dental Medicine, FRANCE

## Abstract

The removal of direct composite veneers, when the retreatment is necessary, represents a challenge to the clinician, since the healthy dental structure must be preserved. Thus, the aim of this study was to compare the accuracy provided by different auxiliary devices during retreatment of direct composite veneers. Seventy-five bovine teeth were prepared for direct composite veneers, scanned (T1), and restored. Specimens were divided into 5 groups for retreatment: conventional high-speed handpiece without auxiliary device (WD); high-speed handpiece with a white LED (WL); high-speed handpiece with an UV light (UL); electric motor and multiplier 1/5 handpiece (EM); and conventional high-speed handpiece using magnifying loupe (ML). After retreatments, other scanning was performed (T2). Changes on dental wear or composite residues areas, as well as, the average between wear and presence of residues were measured. Data were submitted to Kruskal-Wallis and Dunn’s post-test (p≤ 0.05). There were greater areas of wear for ML, being statistically superior to WD and EM groups. The ML presented smaller residues areas, being statistically lower than the WD and EM groups. Regarding the average between wear and the presence of resin residues, additional wear occurred after re-preparation, regardless of the group. Magnifying loupe promoted greater areas of wear and smaller areas of resin residues than conventional high-speed handpiece and electric motor. Both techniques using light accessories did not differ from other ones.

## Introduction

The use of veneers is indicated when the patient has abnormalities such as aesthetic deficiencies and color changes [1, 2]. Porcelain veneers show excellent aesthetic results and predictable longevity of the treatment, while direct composite veneers can be considered as a good conservative option, but with less durability [1]. Failures of direct resin composite veneers occurs because of low resistance to staining and wear related to resin composites [2].

When retreatment is necessary, it is important to highlight the difficulty of removing resin composite due to its similarity with the remaining dental tissue [3]. This procedure represents a challenge for the clinician, since unnecessary dental wear must be avoided during the resin composite removal [4].

Although it is possible to repair resin composite restorations, there are controversial results in the literature regarding bond strength and longevity [5, 6]. The repair consists of a chemical bond between the filler particles and the organic matrix through the use of adhesive systems [5, 6]. Some authors also recommend roughening the surface to prevent microleakage on the new restoration [5]. However, the aged resin composite has less adhesive resistance to a new layer, when compared to recent resins [6]. Another factor to be considered for the success of the repair is the shape of the cavity because box cavity preparations have better retention of restorative material than flat surface such as done for veneer preparations.

Dental market offers different auxiliary devices to aid clinicians during operative procedures [3, 4, 7–10]. One of the most fundamental devices used in Dentistry, the handpiece can enhance the efficiency of everyday dental tasks [7]. Through the years, handpieces have gradually been redesigned and upgraded to become the highly accurate and sophisticated tools they are today [7]. Technological advances continue to improve these indispensable instruments such as light-emitting high-speed handpieces [3, 4]. The most common type of light-emitting high-speed handpieces is with white light-emitting diode (LED) coupled [3, 4]. Other type, recently introduced in the market, is capable of exciting fluorescent particles present in resinous materials. [3, 4, 11–15] These devices emit ultraviolet (UV) light, distinguishing the surface of the dental substrate from the restorative material [3, 4, 11–15].

There are also other options for auxiliary devices in daily restorative treatments. Devices that provide greater control to operator can be used, such as electric motors with multiplier contra-angle that may assist clinicians in achieving greater accuracy in tooth preparation, providing a satisfactory alternative to the air-turbine handpiece [8, 9, 16]. Additionally, surgical loupes have been increasingly popular among dental professionals for their visual and postural benefits [8]. Magnifying loupes allow operators to increase their visual capacity [17], since they compensate for the loss of visual acuity that occurs naturally with age [18].

In this context, the literature is scarce on the use of different auxiliary devices for retreatment of direct resin composite veneers. Furthermore, the present study describes an innovative and precise methodology, through scanning and three-dimensional computerized analysis, to assess changes during the retreatment of direct resin composite veneers. Then, the objective of this *in vitro* study was to evaluate different auxiliary devices in the accuracy of retreating direct resin composite veneers through dimensional changes. The null hypothesis tested was that there would be no statistically significant difference between different auxiliary devices in areas of dental wear and/or presence of resin residues, as well as, in the average between wear and presence of residues for direct veneers retreatments.

## Material and methods

### Samples preparation

This research project was approved by the Animal Use Ethics Committee (CEUA) of Araçatuba School of Dentistry (# 00390–2019). Bovine teeth aged between 24 and 30 months were selected, stunning practices promote good animal welfare, animals feel no pain and become instantly unconscious; after that the throat cut was made immediately after the head is restrained to ensure quick and thorough bleeding of the animal. (Certification Scope: Cattle Slaughterhouse JBS S/A–Andradina/SP–SIF 385, implemented and maintains the Animal Welfare Standards, according to requirements of: AMI–Recommended Animal Handling Guidelines & Audit Guides). For the selection of teeth, the following exclusion criteria were considered: teeth with stains, excessive wear on the incisal third, morphological changes in the clinical crown, and cracks in the enamel. The teeth were mechanically cleaned with sharp periodontal curettes and subjected to prophylaxis with pumice stone and water, with the aid of a brush adjusted at a low-speed handpiece. To avoid bacterial proliferation, clean teeth were stored in a physiological saline solution containing 0.1% thymol and kept in a refrigerator at an approximate temperature of 4°C until the time of the experiment.

Teeth corresponding to color A1 (Vita Classical color scale, VITA Zahnfabrik, Bad Säckingen, Germany) were selected using the spectrophotometer of the intraoral scanning equipment (Trios3, 3Shape, Copenhagen, Denmark). The sample size was calculated with 4 specimens from the pilot study with 0.90 statistical power. The value of 0.13 was obtained for the minimum difference between the averages, and the standard deviation’s average of 0.07. Thus, the minimum sample size was 9 specimens per group. However, 15 teeth were selected per group, considering intra-operator variations.

Teeth had their dimensions reduced in a polishing machine (Politriz, Aropol E, Arotec, Cotia, SP) moved at 100 rpm, with abrasive discs of granulation #100 and under irrigation, up to the dimensions of 10 x 8 mm, simulating the size of a human upper central incisor [19]. Then, the roots were sectioned, reducing the total height (crown and root) to 18 mm, to allow their fixation on the base that was used to perform the procedures.

The specimens were fixed on supports made in a 3D printer (Form 2, Formlabs Inc, Somerville, Massachusetts, United States), with one specimen in each support to make the handling of samples easier. Then, the initial scan (T0) of all specimens was performed [20].

Addition silicone (Elite Transparen, Zhermack SpA, Badia Polesine, RO, Italy) was used for construction of highly transparent matrices that allowed to restore teeth with the same thickness of the resin composite. Teeth were prepared with a conventional high-speed handpiece (without attached light) and with abundant irrigation, by an experienced operator (master student, with 19 years of clinical experience), simulating the characteristics of preparation for direct resin composite veneer. Orientation grooves of 0.3 mm depth were made from cervical to incisal (#4141, KG Sorensen Ind. E Com. Ltda, São Paulo, Brazil). Subsequently, the grooves were merged leading to a whole buccal surface preparation, using a conical diamond tips (#4138, KG Sorensen Ind. E Com. Ltda, São Paulo, Brazil). The cervical outline was a shallow chamfer of 0.1 mm thickness. The cervical and proximal outlines were established in enamel, while parts of buccal surface were defined in dentin. The incisal edge was not performed due to the limitation of the bovine tooth does not have an incisal border.

Then, teeth were scanned again (T1). The average amount of wear between T0 and T1 was measured with specific software (Geomagic Control X, 3D Systems, Rock Hill, South Carolina, USA) to verify the standardization of the preparations. Specimens with a volumetric change of 10% above or below average were excluded from the study. Preparations were standardized with a mean wear value of 0.28 (± 0.07) mm.

The dental surface was conditioned with 35% phosphoric acid (3M ESPE, St. Paul, MN, USA), for 15 seconds on the dentin and 30 seconds on the enamel and then washed for 30 seconds with air/water spray and dried with absorbent paper. An adhesive system (Single Bond Universal, 3M ESPE, St. Paul MN USA) was applied with a micro brush on the dental surface, an air jet was applied to evaporate the solvents and it was photoactivated for 20 seconds with a light-curing device (Valo, Ultradent Products Inc., South Jordan, USA; in Standard mode, 1000 mW/cm^2^). The silicone matrix was used to restore teeth with nanoparticulated resin composite in color A1D (Filtek Z350 XT, 3M ESPE, St. Paul, MN, USA), using the same device cited above, for 40 seconds. The specimens were stored for 30 days in relative humidity at 37°C.

### Randomization and study groups

The specimens were randomly divided into five groups for retreatment procedures: conventional high-speed handpiece and without an auxiliary device (WD), high-speed handpiece with white LED (WL), high-speed handpiece with UV light (UL), electric motor and multiplier 1/5 (EM); conventional high-speed handpiece and use of magnifying loupe 2.5x (ML).

### Specimen re-preparation technique

**Fig 1:**
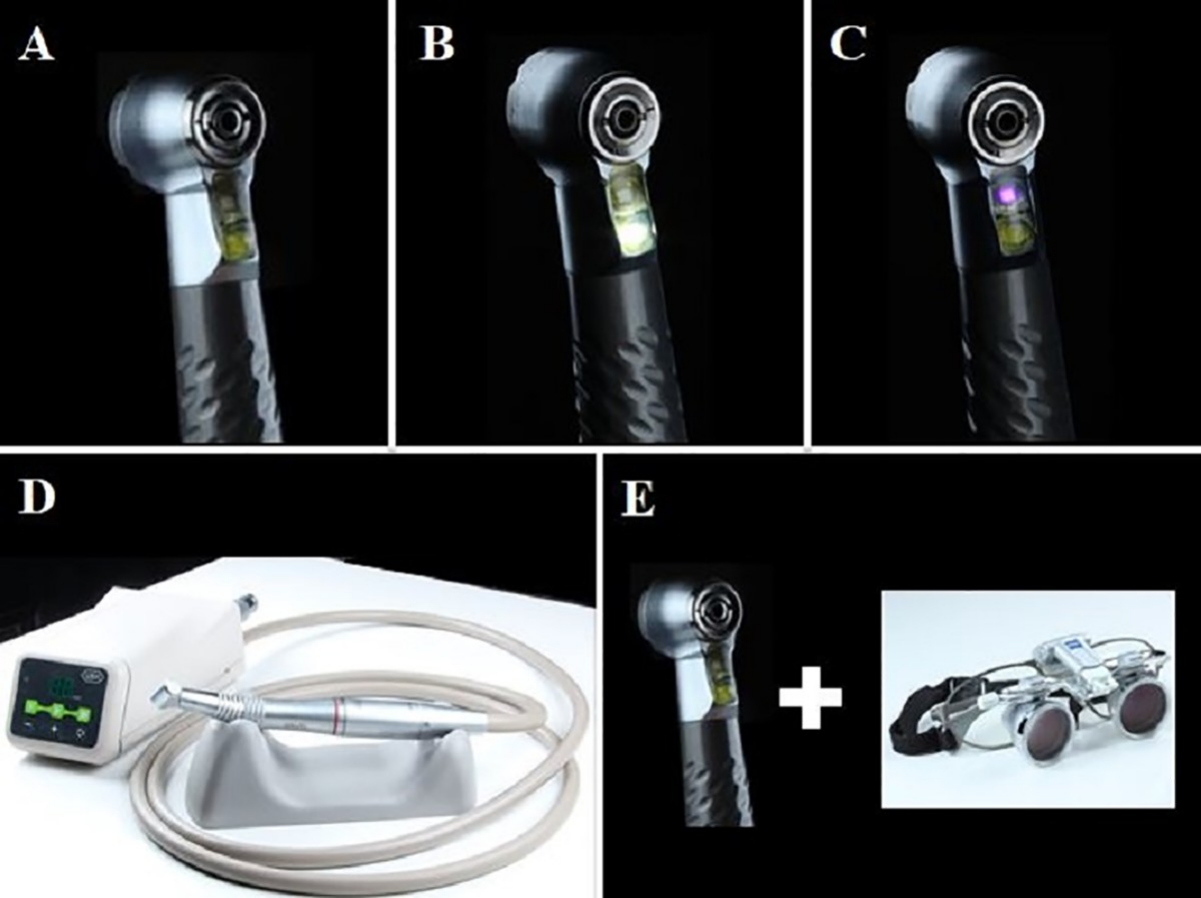
Auxiliary devices according to each group. (a) removal with high-speed handpiece without auxiliary device (WD), (b) removal with high-speed handpiece with a white (WL), (c) removal with high-speed handpiece with an UV light (UL) attached, (d) removal with electric motor and multiplier 1/5 handpiece (EM). (e) removal with conventional high-speed handpiece using magnifying loupe (ML).

The removal of the resin composite and the re-preparation of specimens were performed by another operator (master student, with 2 years of clinical experience), with the same cylindrical diamond tip mentioned before. The high-speed handpiece (Cobra, Gnatus, Ribeirão Preto, SP, Brazil) may be used without light (WD), with only white LED (WL) or with UV light (UL), since the lights are coupled to the handpiece (Fig 1). Only for the EM group, an electric motor (W&H Group, Bürmoos, Austria) was used with a contra-angle multiplier 1:5 (Kavo, Biberach an der Riss, Germany). The duration of each removal procedure was recorded. Then, a new scanning was performed (T2).

### Analysis of dimensional changes

**Fig 2:**
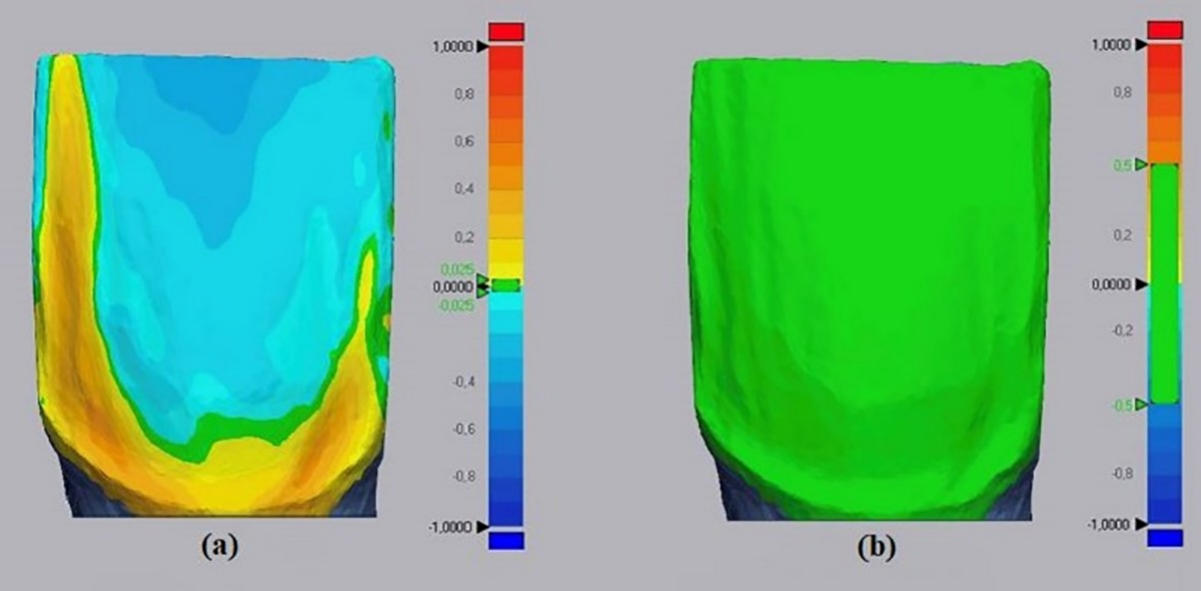
Comparison of tolerance for overlapping digital models in T1 and T2. (a) representative image showing the areas where resin residues are present in yellow, areas of dental wear in blue, and areas where there was no additional dental wear or presence of resin residues in green, with 0.025 mm of tolerance. (b) image showing a 0.5 mm of tolerance, being inadequate to obtain accurate results.

**Fig 3:**
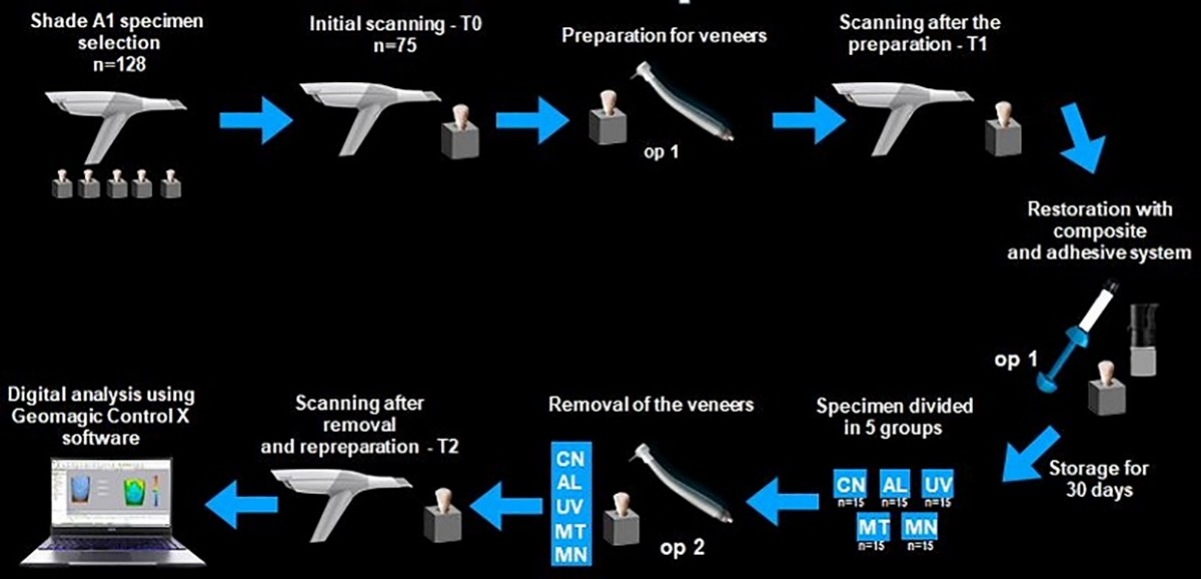
Flowchart of the experiment.

Subsequently, the alterations between T2 and T1 was measured with the same software used for standardization of initial preparations. This software allowed the measurement of dental wear and resin composite residues areas (mm^2^), which are distinguished by different colors (Fig 2). A tolerance of 0.025 mm was established for the overlap in the software to produce accurate results (Fig 2A), with variations described from -1 mm (wear of the remainder) to +1 mm (presence of resinous residues) in the histogram [20, 21]. The average between dental wear and presence of resin residues between T2 and T1 was also calculated. Fig 3 shows the experimental design.

### Statistical analysis

All statistical analyses were implemented with SigmaPlot 12.0 (Systat Software Inc., San Jose, CA, USA). Data normality was assessed by the Shapiro-Wilk test, and homogeneity by Levene’s test.

Data from duration of the re-treatment procedure were analyzed by 1-way ANOVA and the Tukey honestly significant difference post hoc test for multiple comparisons. Data from areas were performed using Kruskal-Wallis and Dunn’s post-test. Statistically significant differences were established with a 5% significance level.

## Results

The mean duration of the removal procedure per restoration was 370 (±75) seconds. No statistical differences were found among groups regarding the time consumption for re-preparation (p≥ 0.05), with following media and standard deviations values in seconds: 357 (±73) for WD, 387 (±88) for WL, 341 (±80) for UL, 358 (±47) for EM, and 408 (±62) for ML.

**Fig 4:**
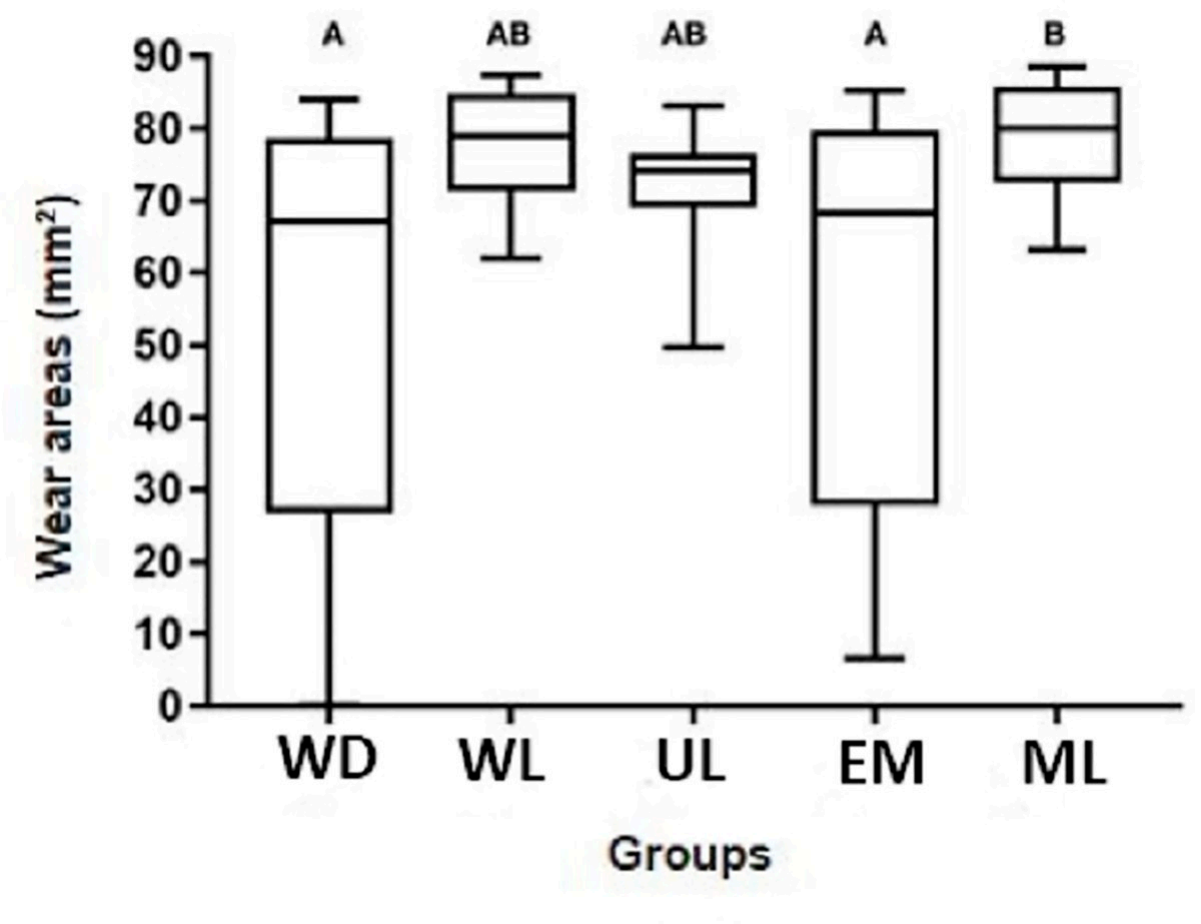
Box plots of the dental wear areas (mm^2^) between T2 and T1 for each group.

**Fig 5:**
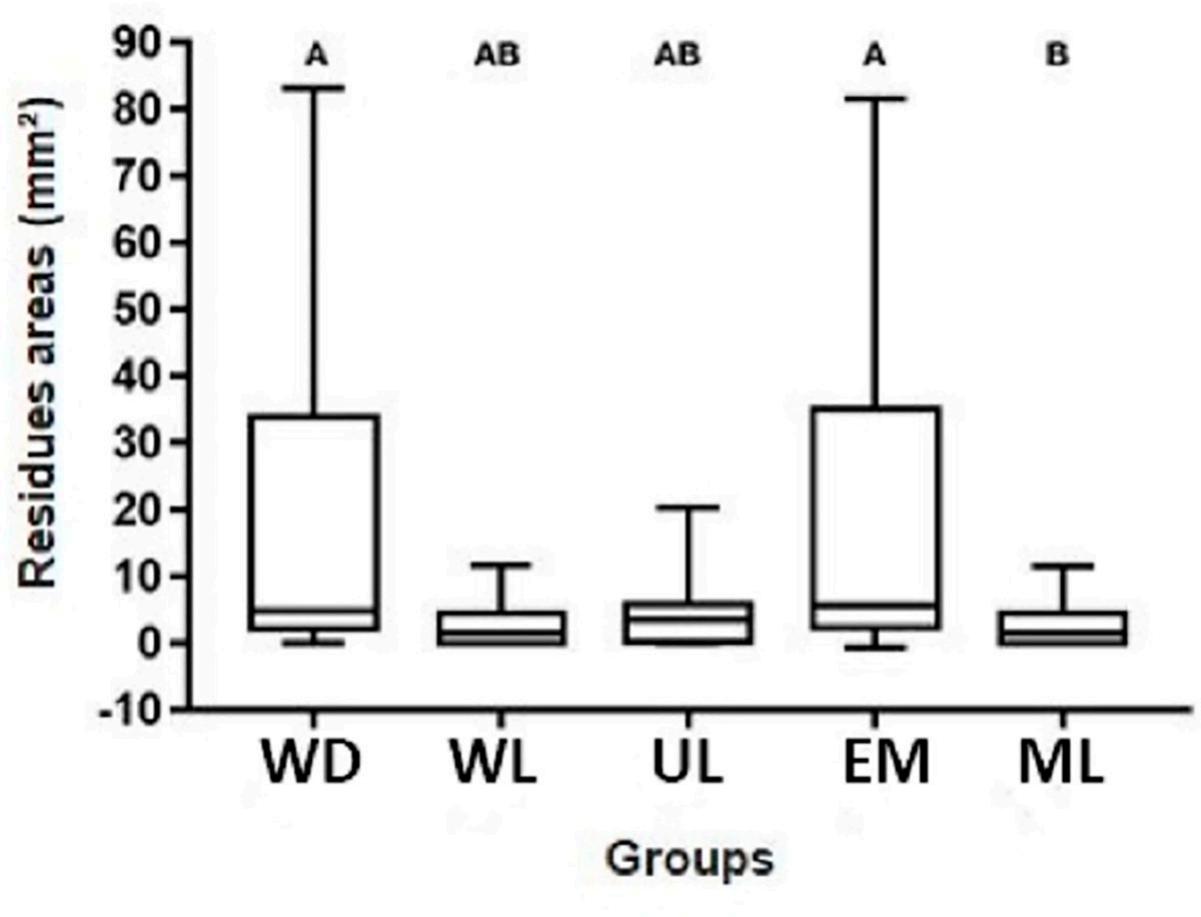
Box plots of the resin residues areas (mm^2^) between T2 and T1 for each group.

Larger areas of dental wear occurred on the surfaces of ML, being statistically similar to WL and UL groups (p≥ 0.05), and higher than WD and EM groups (p≤ 0.05), Fig 4. Regarding the areas of resin composite residues, the opposite occurred since ML group had smaller areas of residues, being statistically similar to the WL and UL groups (p≥ 0.05), and lower to WD and EM groups (p≤ 0.05), Fig 5.

**Fig 6:**
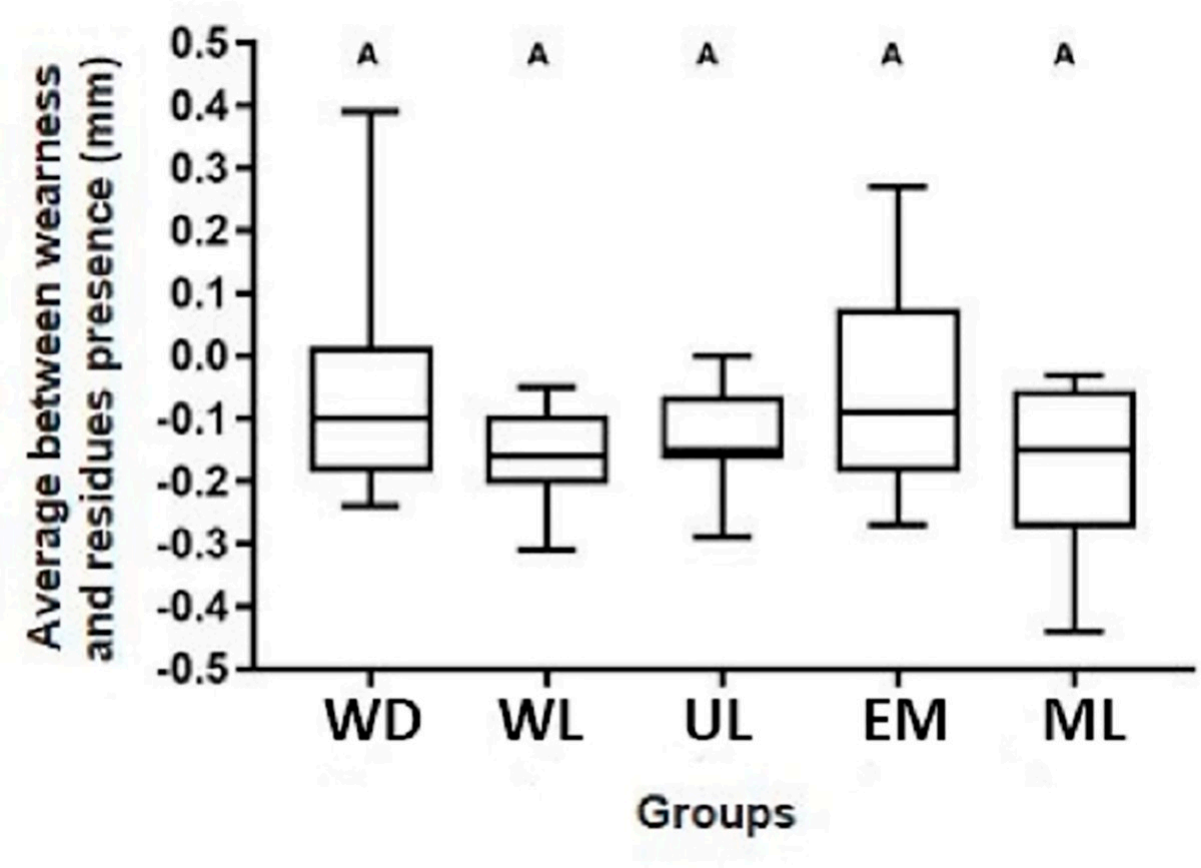
Box plots of the average considering dental wear and resin residues areas (mm^2^) between T2 and T1 for each group.

For all groups, additional wear occurred, with no statistical difference between them concerning the average between wear and the presence of residues after retreatment (p≥ 0.05), Fig 6.

## Discussion

Due to the precision of digital scanning devices, the establishment of new methodologies can expand approaches to evaluate *in vitro* results [20]. Digital technique was investigated to remove resin residues of orthodontic brackets being that the variability attributable to the area and volume measurement techniques was 0.79% and 0.01% for reproducibility, respectively [22].

However, it is important to highlight that these new technologies require specific protocols so that this application reflects the real results and does not produce data with a large margin for interpretation, leading to conclusions with low reliability, such as demonstrated in Fig 2B. Recent studies in operative dentistry have presented methodologies through digital scanning and overlapping of images; however, with the purpose of analyze fluorescence-aided identification technique for posterior restorations with resin composite [13, 21]. Besides, the overlapping graph of digital models was twenty times greater tolerance than that used in the present study [13].

Regarding the re-treatment duration, similar mean duration of the removal procedure per restoration was found in other study for posterior restorations (329 seconds) if compared with our results (370 seconds). It was also found lesser resin removal duration for fluorescence aided composite technique when compared to conventional treatment for one operator, with no significant differences for the other operator [21]. The second operator of the present study aimed at removing composite as completely as possible, having no influence on the re-treatment duration using the different type of devices.

The use of equipment emitting violet light has been described in the literature as an auxiliary method in the selective removal of resin composite restorations [3, 4, 11, 12, 14, 21, 23]. It is known that dentin emits fluorescence in greater intensity than enamel and this is probably due to significant differences in the composition of these substrates, this also applies to resin restorative materials [24, 25]. However, the fluorescence emitted by resin composites can vary in intensity between different trademarks and resins of the same brand, differing according to color and indications [25]. Regarding to auxiliary devices for removing resin composite using light, similar results were found for the dental wear or presence of residues between the WD, WL, UL, and EM groups. These results corroborate with the findings of Klein et al. [13], in which the technique that used white LED also obtained similar values to the group emitting violet light for the presence of residues in class I cavities when the high fluorescence adhesive was not used.

In this study, the resin composite used was chosen because it represents a material that mimics adjacent dental structures, both in color and fluorescence [25, 26], thus making its removal difficult and simulating a clinical condition. However, the similarity between the fluorescence of the resin used in the present study and the dental tissues can be attributed as a factor so that there was no statistical difference between the UL group and other groups for all analyses. Other authors opted to use only resin composites with fluorescence higher than the dental structure when the removal with the aid of fluorescent light was evaluated [3, 14, 15, 21]. This option favored the removal of the resin composite for the removal of orthodontic brackets and also for class I and II restorations, minimizing the presence of residues and reducing the dental wear [3, 14, 21].

Bush et al. [11] suggested the use of UV lighting as an auxiliary method to identify resin residues because they found resin residues for three different degrees of resin fluorescence (highly fluorescent, moderately fluorescent, and weakly fluorescent), after their removal. It is worth mentioning that in a retreatment procedure, the surgeon dentist does not usually have information about the previously resin composite used [27], mainly for restorations performed by another professional.

According to the analysis of dental wear and resin residues areas, the null hypothesis was rejected, since the ML group had a greater area of dental wear and less area of resin residues when compared to the WD and EM groups. It is a fact that the operator must obtain better visualization with the use of oral magnification devices, allowing the dentist to identify resinous residues and perform more precise preparations [28]. The visual acuity of the human eye is about 70 μm, but the vision in a gaseous environment (air), diffraction and refraction reduce to about 150–200 μm [29]. In dentistry, these values can be further compromised by the low luminosity in the oral cavity [30]. It is noteworthy that the operator of the present study aimed to remove the restorative system as completely as possible, justifying the greater wear and tear that occurred in the ML group. The removal of pigmented orthodontic adhesive with the aid of magnifying loupe had advantages in comparison to other methods [28]. In another *in vitro* study, an increase in class I cavity size was found after the removal of resin composite restorations with and without the use of a 2.6X magnifying loupe, finding no significant difference between the groups [31]. Corroborating with the present study, Baumann et al. [17] found that the use of magnifying loupe to remove orthodontic brackets showed less dental wear on the underlying structure, became possibly the visualization and location of the residues on the unprepared enamel surface easier, which has a higher gloss than the resin cement. It is important to highlight that high prevalence of coaxial misalignment among dental professionals was found in a cohort study, in which the use of surgical loupes was evaluated [10]. Then, scientific studies must assist dental professionals in making informed decisions when choosing their magnification equipment and prompt surgical loupe manufacturers to develop more evidence-based products [10].

Electric motor with a 1:5 multiplier provides greater control to the operator during the resin removal procedure, providing less vibration compared to the high-speed handpiece. Also, the electric motor with multiplier is a practical tool that expands and refines the clinical capacity of restorative dentistry under the philosophy of minimally invasive dentistry. The controlled speed rotary instruments were considered more conservative in removing healthy and demineralized dentin, in terms of preparation and depth [32]. However, for the removal of resin composite, the literature is scarce on the use of this device, becoming difficulty comparisons with other studies.

The presence of resinous residues in replacement procedures for a deficient restoration does not significantly affect the adhesion of a new restoration, as long as the appropriate adhesive pretreatment procedures are performed on the surfaces that will receive the new restoration [33]. It is suggested that in deep cavities, resin composite residues should be kept when the proximity of the pulp may be harmful [34]. In addition, it was observed that dentists prefer repair rather than the retreatment of direct restorations in molars compared to premolars and anterior teeth, probably due to the aesthetic question that is more involved in the latter conditions [35].

Regarding to the average between dental wear and presence of resin residues, additional healthy dental structure wear occurs to retreat composite veneers, such as found in another study [31].

As a limitation of this study, the use of resin composite with greater fluorescence than the dental structure may supposedly present different results for the UL group. However, as previously mentioned, the similarity of fluorescence between dental structure and resin composite is necessary for anterior teeth. Another limitation of the present study was that the restorations had no change in color, and probably aged restorations with superficial and interface stains would be better evidenced.

Further research is needed to assess the influence of different operators using currently available auxiliary devices on the treatment duration of resin composite removal. Furthermore, future studies are necessary to improve the technique in retreatments of previous direct veneers, such as the use of laser, since resinous residues can be quickly removed with minimal temperature increase in the pulp and loss of enamel [36]. Further studies are also needed to determine the ocular risks of using a magnifying loupe and the effects of the various types of lights used in dental offices [10, 37].

## Conclusions

Although the use of a dental magnifying loupe has been more effective in removal of resin composite residues during retreatment of direct veneers, it promotes greater dental wear. Regardless of the auxiliary devices used, additional dental wear occurs during retreatment of direct resin composite veneers.
